# The WeanCare nutritional intervention in institutionalized dysphagic older people and its impact on nursing workload and costs: A quasi‐experimental study

**DOI:** 10.1111/jonm.13435

**Published:** 2021-08-17

**Authors:** Milko Zanini, Gianluca Catania, Stefania Ripamonti, Roger Watson, Antonio Romano, Giuseppe Aleo, Fiona Timmins, Loredana Sasso, Annamaria Bagnasco

**Affiliations:** ^1^ Dipartimento di Scienze della Salute Università degli Studi di Genova Genoa Italy; ^2^ Faculty of Health and Social Care University of Hull Hull UK; ^3^ Pharmaceutical and Technical Chemistry Head R&D Healthy Ageing Research Group Cremona Italy; ^4^ UCD School of Nursing, Midwifery and Health Systems University College Dublin Belfield Dublin Ireland

**Keywords:** dysphagia, eating behaviours, elder nutritional physiology, food technology, institutionalized persons, staff workload

## Abstract

**Aim:**

The aim of this study is to explore how a nutritional intervention that improves the biochemical and functional profile of dysphagic older people impacts on nursing workload and costs for nursing homes.

**Background:**

Dysphagic institutionalized older people particularly at risk of malnutrition require more intensive support from nursing staff and higher costs for nursing homes.

**Method:**

This is an open pre–post longitudinal multicentre quasi‐experimental study without a control group.

**Results:**

There is a significant reduction in the number of enemas (from 3.51 to 1.11 enemas), with an average nursing workload reduction from 52 to 16 min per patient every month. Each nurse also spent 20 h less per patient every month spoon‐feeding. This resulted in nursing staff cost savings.

**Conclusions:**

The nutritional intervention led to a significantly better quality of life for the patients manifested through increased independence and social engagement. This reduced workload for nursing staff and costs for nursing home administrators.

**Implications for Nursing Management:**

Sensitive, targeted nutritional interventions have the potential to improve nursing home residents' quality of life and enable a more efficient use of resources. This study revealed reduced workload and cost savings due to less time spent administering enemas and spoon‐feeding, in addition to reduced malnutritional consequences.

## 1 BACKGROUND

As populations age (World Health Organization [WHO], 2015), the requirement for institutionalized care such as nursing home facilities is increasing (Trinkoff et al., 2020). Internationally, there are standards and good practice guidelines that support high quality, fundamental care to nursing home residents (Health Information and Quality Authority [HIQA], 2016). Therefore, there is confidence in fundamental care provision, which is multifaceted and relates to all aspects of daily living (Kitson, 2018). However, in fast‐paced environments, it is recognized that gaps in fundamental care provision can arise (Sasso et al., 2016). There has been limited evidence of specific gaps in nutritional provision (Kalisch & Xie, 2014); however, qualitative studies based on fundamental care by Kitson et al. (2013) have revealed a ‘universal view that food was unappetising’. Mealtime support by health care workers has also been found to be rushed due to time pressures, with little attention to nutritional status (Watkinson‐Powell et al., 2014). At the extreme end, major issues with quality of care for older people in institutional and long‐term settings have in the past raised serious concerns about the lack of attention to nutritional support (Francis, 2013).

In one study, for example, 22.6% of females and 17% of males were classified as malnourished with more than 50% at risk (Donini et al., 2016). Diekmann and Wojzischke (2018) found that the prevalence of malnutrition ranged from 14% to 44% and that one third of nursing home meals needed to be prepared with a modified texture. Moreover, the intake of energy and protein in this population often does not meet the recommended standards reported in the literature, and only one out of every two institutionalized older people manage to finish their meals (Miles et al., 2020).

Particularly, institutionalized older adults with swallowing impairments are at increased risk of malnutrition (Namasivayam & Steele, 2015) because of the difficulties related to preparing their food with the right texture (Massoulard et al., 2011), with the correct amounts of proteins and energy (Pritchard et al., 2014). Of interest, malnutrition is an identified issue in nursing homes even in contexts where the staff perceive residents' nutritional status to be good (ten Cate et al., 2021). Malnutrition is associated with higher levels of co‐morbidity, impaired physical function and reduced cognitive status (Donini et al., 2016). Malnutrition is also clearly linked with the development of pressure ulcers in nursing home residents (Iizaka et al., 2010; O'Brien et al., 2016). Most of the older population are affected by chronic illnesses and co‐morbidities, and if they become malnourished, there is significant impact on health outcomes, co‐morbidities and mortality rates (Wirth et al., 2015). This is problematic, not only because of poor quality of life but there are also associated increased costs for health care systems and organisations (Zanini, Bagnasco, Aleo, et al., 2017).

It is interesting that although the social and cultural aspects of nursing home life are known to be important to residents (Suhonen et al., 2019), the social and cultural aspects of food have received very little attention (Crogan & Evans, 2010). However, a study recently introduced aspects of fine dining to the nursing home setting, including modified‐texture foods (MTFs) with authentic flavours (Zanini, Bagnasco, Catania, et al., 2017). Improved eating experience and increased social interaction have been shown to increase uptake of food (Mathey et al., 2001; Zanini, Bagnasco, Catania, et al., 2017).

The hypothesis of this study is that a better energy and protein intake improves older people's general health conditions (e.g. less time for spoon‐feeding, surveillance, fewer malnutrition consequences and enemas) and consequently also the efficiency of nursing management (Zanini, Bagnasco, Catania, et al., 2017). This is an important point because the rapid ageing of the population increases demand for nursing care and consequently for more efficient nursing management.

The primary objective of this this study was to show how a holistic nutrition intervention (WeanCare) for dysphagic older nursing home residents ensures a more efficient management of nursing staff in terms of less time for spoon‐feeding, surveillance during mealtimes and fewer enemas. The secondary objective of this study was to ensure a better health status and quality of life to nursing home residents.

## 2 METHODS

### 2.1 Design

This is an open pre–post longitudinal multicentre quasi‐experimental study without a control group.

### 2.2 The intervention

The intervention involved the introduction of a personalized modified‐texture nutrition programme called the ‘WeanCare Program’, which consists of meals without nutritional supplementation, where food technology is applied to achieve personalized levels of density, viscosity, texture and particle sizes. It is a novel, low cost, prototype developed by members of the research team, in consultation with the partner company that uses traditional flavoursome Italian recipes that are condensed, dried and packaged for later use. The food is thickened with proteins or naturally occurring bulk items (e.g. collagen), rather than adding additional thickeners such as potatoes. Olive oil is added for taste and to include some fat. These products are low in fat and salt.

The device that produces the MTFs is compact, easy to operate and clean. The finished product although not visually obvious is immediately recognizable, appetizing and palatable upon tasting. Rather than the sticky constitution of thickened food, it is smooth and easy to swallow and distinguishable from the routine ‘soup’ that is created by randomly liquidizing and thickening regular foods. The ‘WeanCare Program’ was included in the routine eating and nutritional plan of all patients with swallowing disorders and with a diagnosis of dysphagia in the 12 nursing homes involved in the project. All patients were fed for 180 days using the WeanCare Program, providing a total of 25,920 MTF meals.

### 2.3 Sample

The sample was selected for convenience by including all the institutionalized dysphagic older people who met our inclusion criteria and lived in the 12 nursing homes that participated in the present study. Sampling size adequacy was based on a literature analysis based on previous similar studies, where the sample size refers to a before–after study verified through a paired *t* test (Chow et al., 2007). The result of the sample size calculation was *N* = 60.0191, which was rounded up to 61 in relation to the participants' dropout risk. For this calculation, we used the value of the total proteins, which was used both for the present study and for our previous study (Zanini, Bagnasco, Catania, et al., 2017).

The nursing homes were selected through convenience sampling by personally contacting via email and then by phone the medical directors of the nursing homes and asking them if in principle they were willing to participate in this study.

After obtaining the authorization to conduct the nutrition intervention from the medical directors of the nursing homes, consent was obtained from the patients or their legal proxies. Patients were included if they were being fed with blended soft foods following the diagnosis of dysphagia, which had been either reported in the medical record or confirmed by a physician of the nursing home. Therefore, all dysphagic patients over the age of 65 years, with low co‐morbidity levels (Cumulative Illness Rating Scale < 6), and whose diet was consistent with Levels 3 and 4 of the International Dysphagia Diet Standardisation Initiative Framework (IDDSI, 2019) were included in this study.

Patients were excluded if they presented high levels of co‐morbidity (Cumulative Illness Rating Scale > 6), clinical instability, chronic or cancer diseases, severe dysphagia (Dysphagia Outcome and Severity Scale ≥ 2), percutaneous endoscopic gastrostomy feeding, orogastric tubes and terminally ill patients.

### 2.4 Data collection

In addition to the data required for the inclusion criteria, our data sets also included information from the patients' previous medical records. The indicators used in the present study included information routinely collected by nursing homes. Data were collected for 6 months, from 8 January 2018 to 11 March 2019. Anthropometrical, biochemical, nutritional and functional parameters were collected at Time 0 and, prospectively, every 3 months after implementing the dedicated food programme for a total of three experimental evaluations (T0 = starting point, T3 = after 3 months, T6 = after 6 months, end of the study).

Serologic (lymphocyte count normal range: 1.40–3.50/μl), anthropometric (albumin normal range: 3.6–5.5 g/dl) and nutritional performance (total protein normal range: 6.4–8.3 g/dl) measures were collected to evaluate the enhancement of these parameters and their relationship with patients' well‐being (Keller, 2019). Serologic data were used to evaluate whether daily nutritional intake was adequate in relation to the meals offered and were correlated with the patients' inflammatory status.

The menus were prepared according to the patients' values about food and reproduced the regular menus of the nursing home, recreating a familiar environment also for people with a MTF diet. In addition to the conditions of general malnutrition, we also evaluated some general conditions related to the patients' quality of life (e.g. participation in socialization activities, ability to eat on their own, amount of meal consumption, need to be spoon‐fed and administration of enemas). Nutritionists, nurses and researchers were involved in data collection.

Difficulty with mealtimes was measured using the Edinburgh Feeding Evaluation in Dementia (EdFED) scale, which has been translated into and validated in Italian (Bagnasco et al., 2015). The EdFED scale, first developed by Watson (1994), has been described as the only validated scale for mealtime difficulty in older people available internationally (Alzheimer's Disease International, 2014). It has been extensively used in international studies of nutrition and eating in older people (Lin et al., 2010; Lin et al., 2011). The scale consists of 10 items enquiring about mealtime difficulty (e.g. ‘Does the patient refuse to swallow?’) scored on a 3‐point Likert scale 0 (*never*), 1 (*sometimes*) and 3 (*always*). Six of the items form a single factor composed of items referring specifically to the behavioural aspects of dementia (Watson & Deary, 1997), and these items form a hierarchy of difficulty (Watson, 1996). The remaining items refer to nursing care and related observations.

### 2.5 Ethical approval

Ethical approval was obtained by the Liguria Regional Ethics Committee (ID 11116 ‐ no. 677/2020).

## 3 RESULTS

A total of 72 (15 males and 57 females) older people were recruited in this study. Of these, 70 suffered from moderate to severe cognitive impairment, based on the MDS Cognitive Performance Scale (Morris et al., 1994). Initially, the sample consisted of 78 individuals, but five died and one was discharged, so the final sample consisted of 72 individuals (Table 1).

**TABLE 1:** Sample characteristics

Sample characteristics	
Initial sample recruited	78
Dropped out	6 (5 died and 1 was discharged)
Final sample size	72
Males	15 (20.83%)
Females	57 (79.17%)
Mean age (years)	87 (SD 6.3)
Moderate to severe cognitive impairment (according to the MDS Cognitive Performance Scale)	70 (97.2%)
Participated in social activities	36 (51%)
Ate regularly	63 (87%)
Finished their meals	51 (71%)

In the present study, we measured total proteins, albumin and lymphocytes as indirect indicators of malnutrition (Evans et al., 2021; Sergi et al., 2006), considering the inflammatory status of the patients detected by the nursing homes by means of blood tests.

**FIGURE 1:**
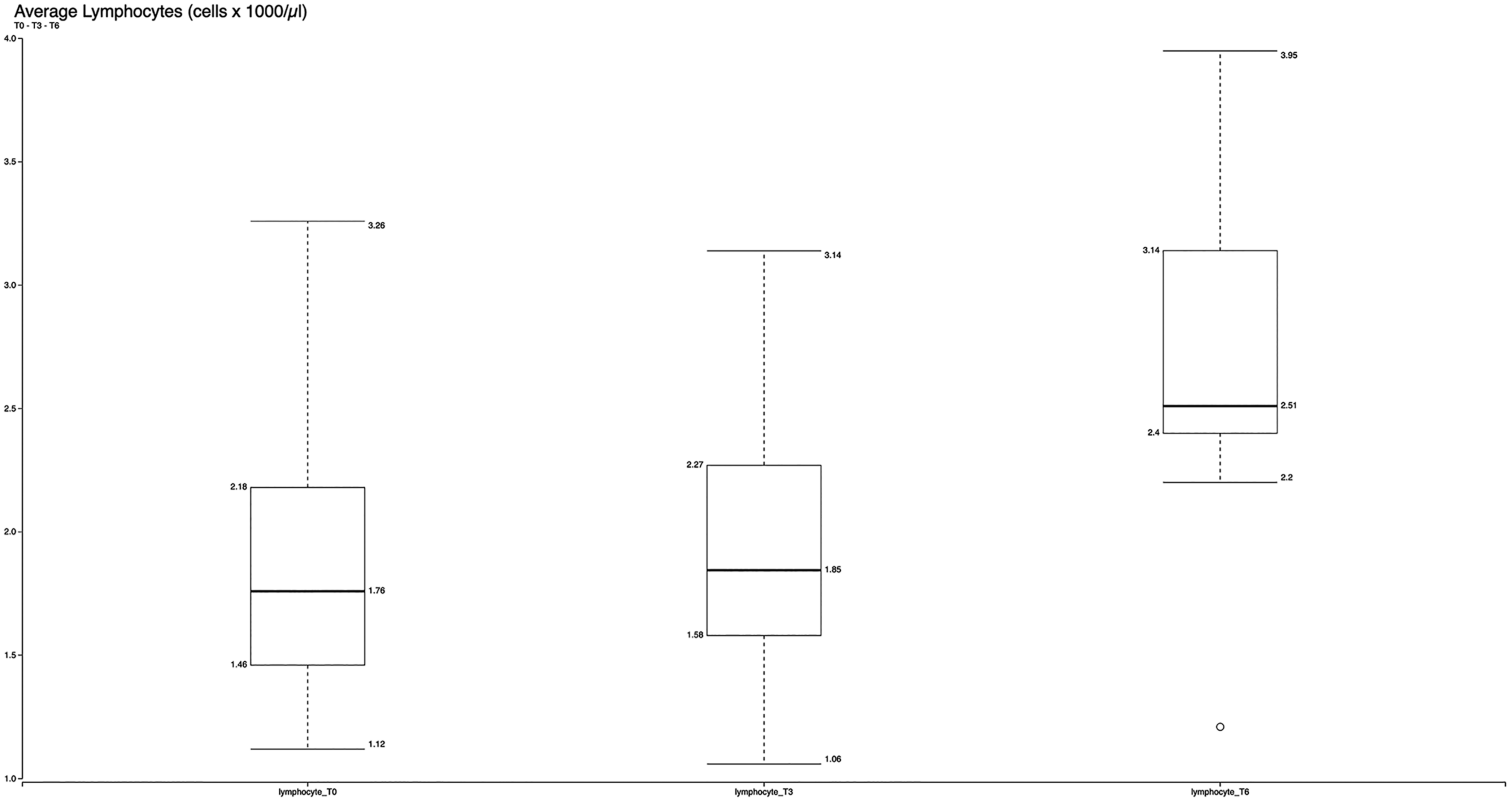
Lymphocyte total mean count over 6 months in a total population of 72 older people: from T0 = 1.99/μl (SD 0.67) to T3 (after 3 months) = 2.08/μl (SD 0.78) and to T6 (after 6 months) = 2.87/μl (SD 0.77); *p* > .0001

The lymphocyte total mean count increased from 1.99/μl (SD 0.67) at Time 0 (T0) to 2.08/μl (SD 0.78) after 3 months (T3) and to 2.87/μl (SD 0.77) after 6 months (T6) (normal range: 1.40–3.50/μl). The two‐tailed *p* value was less than .0001, and the 95% confidence interval of this difference changed from −1.1203 to −0.6397 (Figure 1). A higher lymphocyte total mean count showed that after the 6‐month WeanCare nutritional intervention, the nursing homes residents had a lower risk of infection and more stable health conditions in general.

**FIGURE 2:**
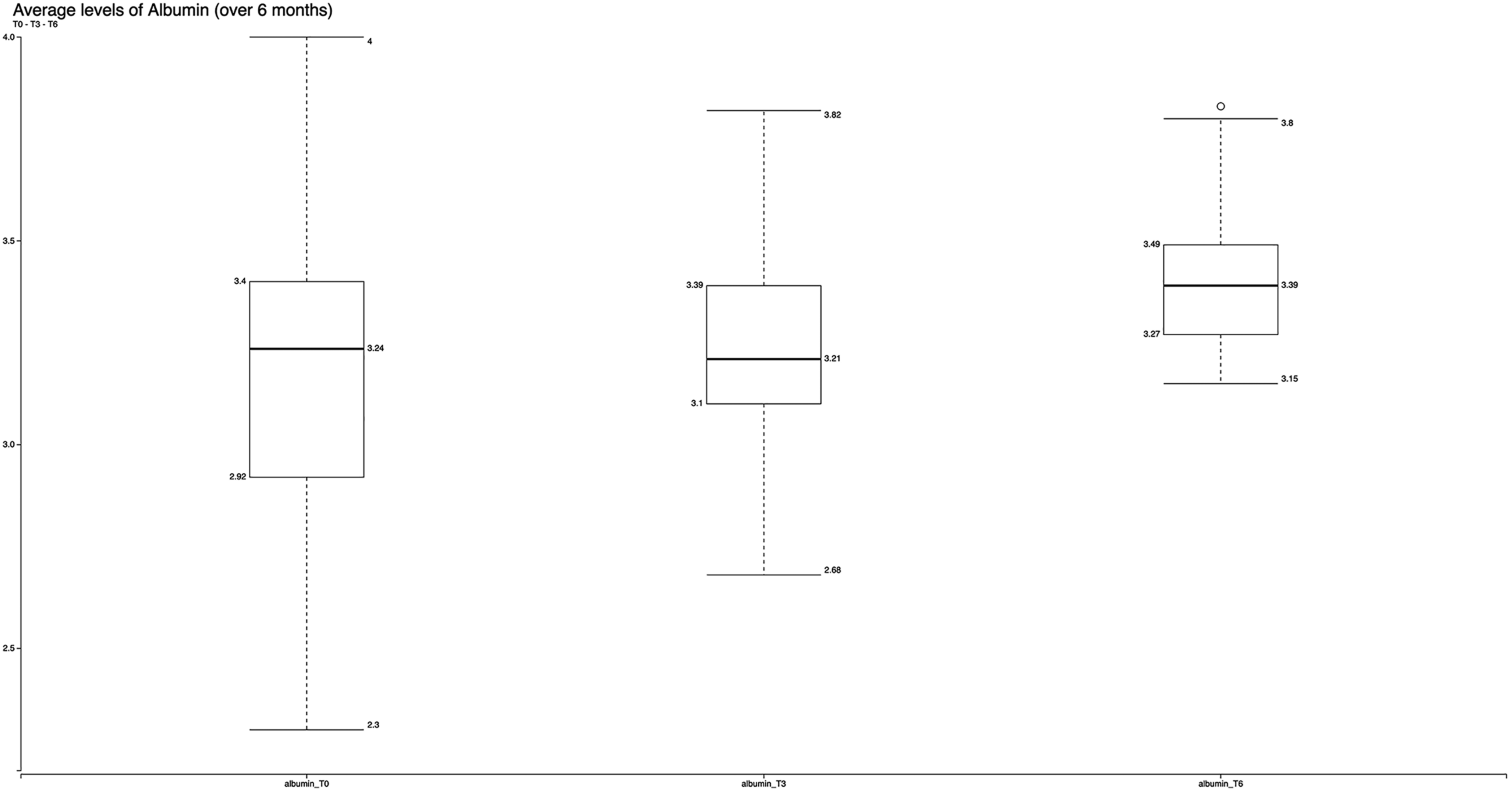
Increase of the mean levels of albumin over 6 months in a total population of 72 older people: from 3.14 (SD 0.4) at T0 to 3.42 (SD 0.2) at T6

Body weight and the related body mass index (BMI) remained almost the same after 6 months, and no side effects were reported. The mean weight increase was equal to 1.6 kg (SD 1.92 kg). Significant correlations were found between higher levels of albumin, which increased from 3.14 g/dl (SD 0.4) at T0 to 3.42 g/dl (SD 0.2) (normal range: 3.6–5.5 g/dl) after 6 months (T6) and the healing of pressure wounds (0.01) (Figure 2). All the pressure wounds had already healed after 3 months since the start of the nutritional intervention.

**FIGURE 3:**
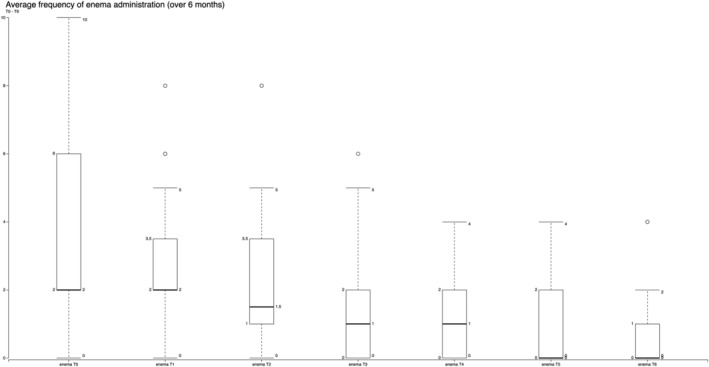
Mean reduction of frequency of enema administration over 6 months in a total sample of 72 older people: from 3.51 (SD 2.49) at T0 to 1.11 (SD 1.89) at T6. Two‐tailed *p* value < .0001

The overall increased consumption of meals throughout the day increased the amount of fluid intake, which together with the introduction of vegetable fibres enabled to drastically reduce the number of enemas performed every month. The mean amount of time needed to administer an enema in these patients was approximately 15 min (±2). Following our intervention, for each patient, we passed from an average of 3.51 (SD 2.49) at T0 to 1.11 (SD 1.89) enemas after 6 months (T6) (Figure 3), leading to significant results in terms of reduced workload, passing from an average of 52.71 (SD 37.43) min per patient every month at T0 to 16.67 min per patient every month (SD 28.43) after 6 months. In other words, at T0 only 8 (11.11%) patients did not require enemas, whereas at T6, this number rose to 47 (65.28%). This led to a better quality of life for the patients, reduced workload for health care staff and savings for the nursing homes due to the reduced use of enemas.

Therefore, patients' general conditions related to clinical data associated to malnutrition significantly improved over time, but the most interesting findings were related to their eating behaviours. After 6 months, six (8.33%) additional patients started eating their meals regularly. Moreover, in the same timeframe, eight (11.11%) patients started eating all their meals from *never* to *always*, and another five (6.94%) patients started eating all their meals from *sometimes* to *always* (Table 2).

**TABLE 2:** Patients who ate all their meals after 6 months

Ate all their meals at T0	Ate all their meals at T6	Frequency	Percentage
Never	Never	3	4.17
Never	Sometimes^a^	1	1.39
Never	Yes	8	11.11
Sometimes^a^	Sometimes^a^	1	1.39
Sometimes^a^	Always	5	6.94
Always	Always	54	75.0

^a^ Up to 10% of the time.

Moreover, after 6 months, 13 patients (18.06%) no longer needed to be spoon‐fed (Table 3). Because about 20 min were required to spoon‐feed each patient, this result enabled each nurse to save 40 min a day, equal to a total of 20 h a month.

**TABLE 3:** Patients needing to be spoon‐fed after 6 months

Spoon‐fed at T0	Spoon‐fed at T6	Frequency	Percentage
No	No	24	33.33
Yes	No	13	18.06
Yes	Yes	35	48.61

**FIGURE 4:**
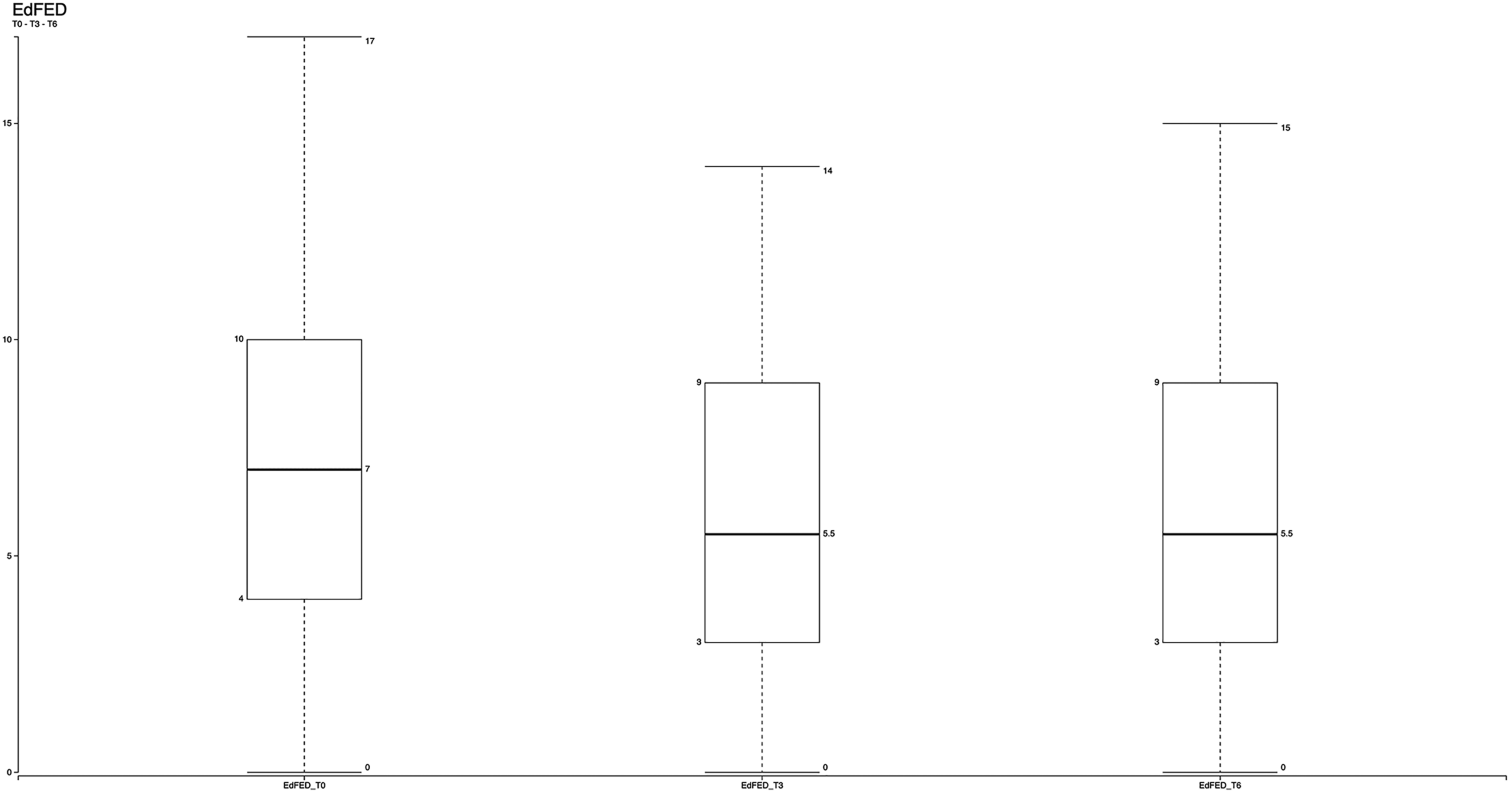
Box plot showing how the average levels of difficulties during feeding using the Edinburgh Feeding Evaluation in Dementia (EdFED) score dropped from 7.27 (Time 0) to 5.57 (Time 6) after 6 months in our total sample of 70 older people. Two‐tailed *p* value = .03

The patients' reduced self‐feeding difficulties, from 7.27 (Time 0) to 5.57 after 6 months—measured using the EdFED Scale (Watson, 1994)—significantly contributed to reducing the patients' nutritional risk (Figure 4).

This also resulted in 15 (20.83%) patients who started taking part in socialization activities after 6 months (Table 4) and therefore no longer required to have their meals served in their own rooms, with a reduction of staff required for surveillance during mealtimes.

**TABLE 4:** Patients who took part in socialization activities after 6 months

Participation in socialization activities at T0	Participation in socialization activities at T6	Frequency	Percentage
No	No	20	27.78
No	Yes	15	20.83
Yes	Yes	37	51.39

## 4 DISCUSSION

### 4.1 Food acceptance, socialization and surveillance

Murphy et al.'s (2017) focus groups with family and carers of nursing home residents highlighted the importance of the social and relational aspects of eating. Local interventions can be targeted and focused on improving both the social aspect of eating (Regional Geriatric Program of Ontario, 2010; Zanini, Bagnasco, Aleo, et al., 2017) and the eating experience (Chapman et al., 2015; Zanini, Bagnasco, Catania, et al., 2017). In terms of nursing management, there are important practical benefits linked to the social aspects of eating because the consequence of higher levels of autonomy in this population also has a significant impact on reducing workload and time that nurses and other health care workers dedicate to spoon‐feeding and surveillance during mealtimes. When nursing home residents do not participate in socializing activities, they remain in their own room, which is often on another floor, and this requires someone nearby to keep an eye on them. Instead, when patients sit together and participate in socializing activities, surveillance can be ensured by fewer staff, thus reducing staffing costs (Zanini, Bagnasco, Aleo, et al., 2017).

### 4.2 Less spoon‐feeding

The WeanCare modified‐texture nutritional intervention significantly reduced the levels of swallowing exhaustion and impairment caused by the prolonged difficulty trying to tolerate excessive volumes of foods and fluids (Painter et al., 2017; Serra‐Prat et al., 2011) in our sample of institutionalized older people affected by dysphagia. Our participants improved their individual ability to swallow and their ability to eat on their own. This translated into more efficient use of staff time, because these patients started eating autonomously and no longer required to be spoon‐fed, as also observed by Keller et al. (2017).

Moreover, taste and texture improve appetite and intake. One of the main differences between the WeanCare intervention of the present study and other previous similar interventions (Farrer et al., 2016; Okkels et al., 2018) was that the patients appreciated and recognized the meals because they were the same to what they were traditionally used to eating. In our study, all this enabled to significantly reduce the number (−13) of residents who required to be spoon‐fed, thus reducing the workload for nurses and enabling them to dedicate their time to other nursing activities. This in terms of nursing management translates in more efficient use of staff time (Barrientos‐Trigo et al., 2018).

### 4.3 Better nutrition, better general conditions and less workload on nurses

Another important aspect of this intervention is the palatability of the food. When the nursing home residents recognized the food, they enjoyed it more and ate more, something which had been demonstrated also in other studies (Zanini, Bagnasco, Catania, et al., 2017). Preventing malnutrition requires a balanced, nutritious diet that is also appetizing (Blössner & de Onis, 2005) and contributes to better general health conditions, well‐being and recovery (Drevet et al., 2014; Gordon et al., 2014). From a nursing management perspective, it is important that patients eat regularly all their meals because this improves their daily intake of liquids, proteins, lipids and carbohydrates, enabling them to feel better and healthier and therefore require fewer caring activities. Considering the growing numbers of older nursing home residents and co‐morbidities, eating dependence is an increasing concern (Miles et al., 2020; Vikström et al., 2021; Zanini, Bagnasco, Catania, et al., 2017). Additionally, malnutrition in older people can give rise to significant negative effects such as infections, dehydration, wound healing problems, falls, risk of developing pressure ulcers and overall increased morbidity and mortality (Landi et al., 2016). Malnutrition also increases older people's weakness and reduces alertness, affecting their quality of life, such as the ability to walk, socialize and perform activities of daily living, such as eating, personal hygiene and getting dressed (Bassola et al., 2020), all which impact on the workload of nursing staff.

### 4.4 Fewer enemas, more efficient use of staff time and resources

The nursing home residents' improved ability to eat all their food also increased their fluid intake, which consequently reduced the need to resort to enemas to facilitate bowl movement and defecation. The reduced requirement for enemas based on an individualized diet that included fibres and hydration that reduced constipation was also a significant finding, as this not only improved quality of life for residents, by restoring a greater sense of normality, but similarly to the findings of another previous study (Palese et al., 2010), nurses made a more efficient use of their working hours and reduced costs related to purchasing and administering enemas.

## 5 LIMITATIONS

The sample size was limited and based on convenience sampling, therefore more methodologically rigorous studies, such as randomized controlled trials, are needed in the future. It was difficult to access additional diagnostics because the nursing homes were obliged to manage patients with predefined costs. The Covid‐19 pandemic significantly influenced data collection (restricted access) and patients' survival rates. A strength of this study was that all the nursing home staff realized how important this nutritional approach was for its organisational management.

## 6 CONCLUSIONS

The provision of appetizing meals with a texture that is modified according to individual swallowing ability, ensuring at the same time the right amount of energy and protein, significantly improved the health conditions especially in patients with swallowing disorders. From a nursing management perspective, this nutritional intervention produced significant results in terms of reduced workload, more efficient use of nursing staff and less costs.

Practice and policy in this fundamental aspect of care need to be advanced. Research on the benefits of nutrition in older people needs to be increased, and the nursing home managers need to adjust their emphasis on this fundamental aspect of care (Kitson, 2018). Nursing managers have the moral, professional and ethical responsibility to pay more attention to the clinical, human, social and organisational benefits of nutrition.

## 7 IMPLICATIONS FOR NURSING MANAGEMENT

Nursing interventions that are targeted to improving nutrition in older people living in nursing homes can have a significant impact on health care outcomes and consequently also on the nursing management. Malnutrition among institutionalized older people is a substantial challenge, likely to increase as the older population grows. Nurses have a key role in this fundamental aspect of care, not only through careful assessment but also through nutritional care interventions aimed at improving the general health conditions of older people (Bonetti et al., 2013). In addition to improvements across many key performance indicators, attention to nutritional status in this study resulted in a more efficient use of nurses' working hours and cost savings due to reduced time for spoon‐feeding, surveillance, fewer consequences linked to malnutrition and fewer enemas. Paying attention to this fundamental aspect of care, and raising awareness about dietary needs, both physical and social, will also help to prevent major lapses in care (Francis, 2013) and serious consequences arising for nurse managers as a result (Hayter, 2013).

## CONFLICT OF INTEREST

There is no conflict of interest.

## AUTHOR CONTRIBUTIONS

AB, GC, MZ, AR and LS are involved in study conception or design. AB, AR, GC, MZ, AR and SR collected the data. RW and MZ analysed and interpreted the data. AB, MZ and LS supervised the study. MZ, GA and FT wrote the manuscript. MZ, AB, LS and FT are involved in critical revisions for important intellectual content. All authors approved the final version.

## ETHICAL APPROVAL

Ethical approval was obtained by the Liguria Regional Ethics Committee (ID 11116 ‐ no. 677/2020).

## Data Availability

The authors elect to not share data.
